# Astragaloside IV ameliorates diabetic nephropathy by modulating the mitochondrial quality control network

**DOI:** 10.1371/journal.pone.0182558

**Published:** 2017-08-02

**Authors:** Xinhui Liu, Wenjing Wang, Gaofeng Song, Xian Wei, Youjia Zeng, Pengxun Han, Dongtao Wang, Mumin Shao, Juan Wu, Huili Sun, Guoliang Xiong, Shunmin Li

**Affiliations:** 1 Department of Nephrology, Shenzhen Traditional Chinese Medicine Hospital, Guangzhou University of Chinese Medicine, Shenzhen, Guangdong, China; 2 Department of Pathology, Shenzhen Traditional Chinese Medicine Hospital, Guangzhou University of Chinese Medicine, Shenzhen, Guangdong, China; 3 Department of Nephrology, Zhejiang Provincial People’s Hospital, Hangzhou, Zhejiang, China; University of California Los Angeles, UNITED STATES

## Abstract

The aim of this study was to investigate the effect and possible mechanism of Astragaloside IV (AS-IV) on retarding the progression of diabetic nephropathy (DN) in a type 2 diabetic animal model, *db/db* mice. Eight-week-old male *db/db* diabetic mice and their nondiabetic littermate control *db/m* mice were used in the present study. AS-IV was administered to the *db/db* mice by adding it to standard feed at a dose of 1g/kg for 12 weeks. Renal injury was assessed by urinary albumin excretion (UAE) and Periodic acid-Schiff staining. The protein expression levels of mitochondrial quality-control-associated proteins were evaluated using Western blotting and immunohistochemical staining analysis. At the end of the experiment, *db/db* mice showed overt renal injury, as evidenced by increased UAE, increased urinary N-acetyl-β-D-glucosaminidase (NAG), expansion of mesangial matrix, and increased renal tubular area. AS-IV administration significantly reduced UAE and urinary NAG and ameliorated the renal pathologic injury seen in *db/db* mice. Furthermore, the expression of dynamin-related protein 1 (Drp-1), mitochondrial fission protein 1 (Fis-1), and mitochondrial fission factor (MFF), the main regulators of mitochondrial fission, was significantly increased in *db/db* mice. Moreover, PTEN-induced putative kinase 1 (PINK1)/Parkin-mediated mitophagy was abnormally activated in *db/db* mice. AS-IV significantly reduced renal Drp-1, Fis-1, and MFF expression and downregulated PINK1/Parkin-mediated mitophagy in *db/db* mice. However, mitochondrial biogenesis and mitochondrial fusion-associated protein levels were not significantly different between *db/m* and *db/db* mice in our study, with or without AS-IV treatment. In conclusion, administration of AS-IV could retard DN progression in type 2 diabetes mice, which might be associated with restoration of the mitochondrial quality control network.

## Introduction

According to the International Diabetes Federation (IDF), the estimated diabetes prevalence for adults between the ages of 20 and 79 worldwide for 2015 was 415 million; the disease is expected to affect 642 million people by 2040 [1]. Diabetic nephropathy (DN) is a progressive microvascular complication arising from diabetes and is the leading cause of chronic kidney disease (CKD) and end-stage renal disease (ESRD) worldwide [2]. Although the molecular mechanisms implicated in the pathogenesis and progression of DN remain unclear, increasing evidence indicates that disturbances in mitochondrial homeostasis might be important in the development and progression of DN [3].

Mitochondria are dynamic organelles that play many essential roles in the regulation of energy metabolism, signal transduction, cell differentiation, cell proliferation, and cell death [4]. The kidney is a highly aerobic organ and is rich in mitochondria. Therefore, kidneys are exquisitely dependent upon, and susceptible to, being damaged by mitochondria. A growing body of evidence shows that mitochondrial dysfunction plays a pivotal role in the pathogenesis of various kidney diseases [5]. Mitochondrial homeostasis is maintained by a mitochondrial quality control network, including at least mitochondrial biogenesis, mitochondrial fission and fusion, and mitochondrial autophagy (mitophagy) [6]. Emerging evidence suggests that disturbances in the mitochondrial quality control network might be important in DN pathogenesis [7]. However, the alteration of mitochondrial quality control regulation in the kidney of a type 2 diabetes animal model is not well defined and needs to be investigated.

**Fig 1:**
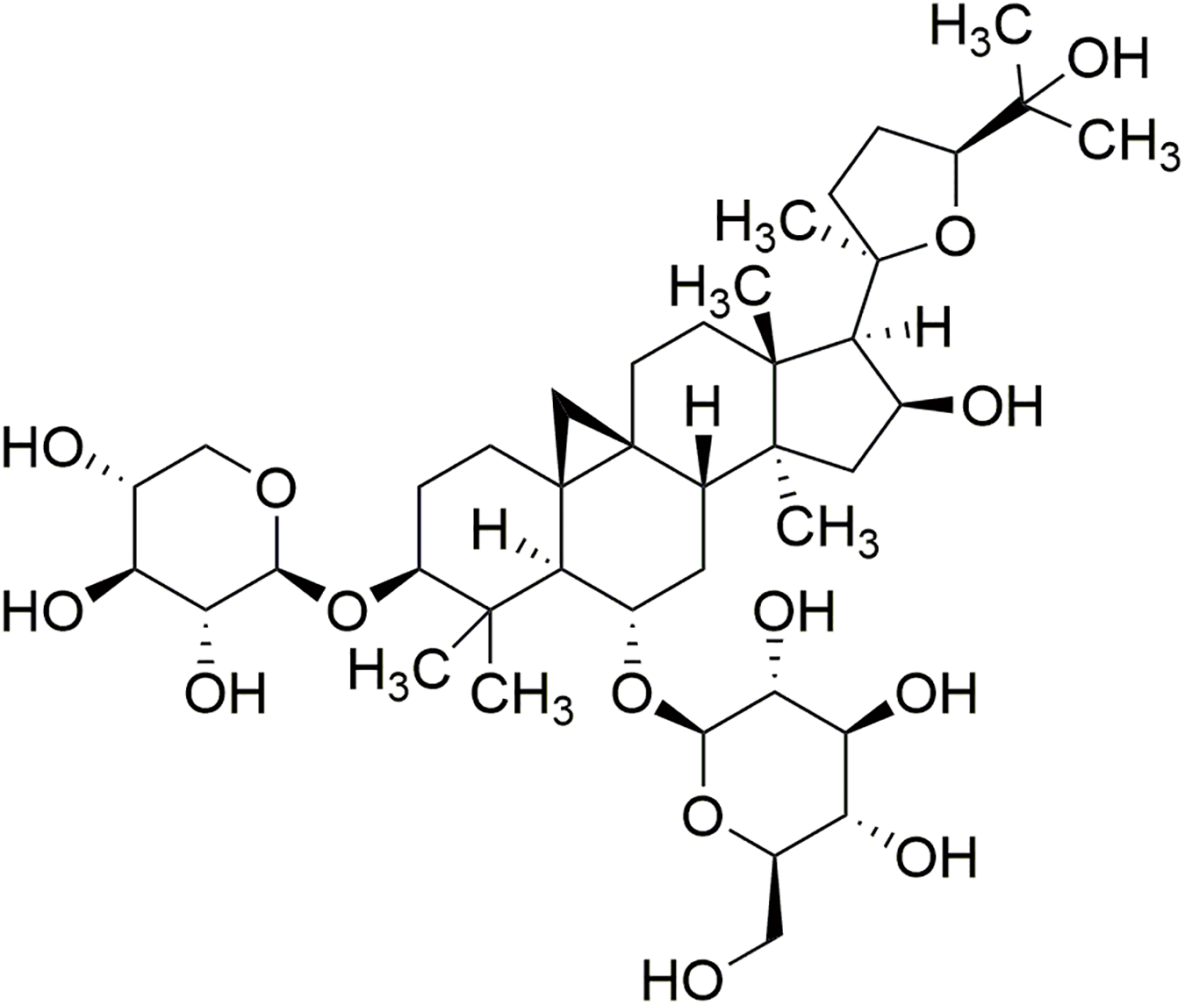
Structure of Astragaloside IV (AS-IV).

Astragaloside IV (AS-IV) is a lanolin alcohol-shaped tetracyclic triterpenoid saponin with high polarity (Fig 1) and is one of the major and active components of *Astragalus membranaceus Bunge*, an herb widely used in traditional Chinese medicine for tonifying *Qi*. In recent decades, diverse pharmacological activities of AS-IV have been demonstrated, including anti-inflammatory, anti-oxidative stress, anti-apoptosis, and anti-fibrosis activities, both *in vitro* and *in vivo* [8]. Recent studies have shown that AS-IV administration ameliorates DN in streptozotocin (STZ)-induced diabetic rats via an anti-inflammatory mechanism [9], inhibits endoplasmic reticulum stress [10], and protects podocytes [11,12]. However, the effect and mechanism of AS-IV on DN induced by type 2 diabetes remain unknown.

In the present study, we evaluate the status of the kidney mitochondrial quality control network and explore the effect of AS-IV on ameliorating DN in an experimental mouse model of type 2 diabetes. Our results suggest that the renoprotective effect of AS-IV on DN in the state of type 2 diabetes might be associated with the modulation of the mitochondrial quality control network, which was deranged in *db/db* mice.

## Materials and methods

### Chemicals and antibodies

AS-IV (C_41_H_68_O_14_, molecular weight = 784.97, CAS no. 84687-43-4) was purchased from ConBon Biotechnology, Chengdu, China. The primary antibodies included rabbit anti-peroxisome proliferator-activated receptor-γ coactivator-1α (PGC-1α), rabbit anti-mitochondrial fission protein 1 (Fis-1), rabbit anti-nuclear respiratory factor 1 (NRF-1) (Santa Cruz Biotechnology, Santa Cruz, CA, USA), rabbit anti-dynamin-related protein 1 (Drp-1), rabbit anti-mitofusin 2 (Mfn-2), rabbit anti-voltage-dependent anion channel (VDAC) (Cell Signaling Technology, Beverly, MA, USA), mouse anti-optic atrophy 1 (OPA-1) (BD Biosciences, San Jose, CA, USA), rabbit anti-PTEN-induced putative kinase 1 (PINK1) (Gene Tex, San Antonio, TX, USA), rabbit anti-Parkin (phospho S65), mouse anti-mitofusin 1 (Mfn-1) (abcam, Cambridge, MA, USA), mouse anti-β-actin, mouse anti-Parkin, rabbit anti-light chain 3 (LC-3) (Sigma-Aldrich, St Louis, MO, USA), mouse anti-heat shock protein 60 (HSP-60), and rabbit anti-mitochondrial fission factor (MFF) (proteintech, Wuhan, China). Horseradish peroxidase (HRP)-conjugated anti-mouse IgG and HRP-conjugated anti-rabbit IgG were purchased from Life Technologies (Carlsbad, CA, USA).

### Animals and experimental treatments

All animal procedures were conducted with protocol approval from the Ethics Committee of Shenzhen Traditional Chinese Medicine Hospital, Guangzhou University of Traditional Chinese Medicine (Shenzhen, China), and all efforts were made to minimize animal suffering. Male *db/db* diabetic mice in the C57BLKS/JNju background and their nondiabetic littermate control *db/m* mice (8 weeks old) were obtained from the Model Animal Research Center of Nanjing University. The mice were housed in a specific pathogen-free (SPF) animal facility under a 12-hour light/12-hour dark cycle with stable temperature (22°C-25°C) and humidity (40%-70%) and were allowed free access to sterile pelleted feed and distilled water during the experiments. After one week of acclimatization, urine samples of the *db/db* mice were collected for 24 hours in metabolic cages (Tecniplast, Italy). According to values of 24-hour urinary albumin excretion (UAE), all *db/db* mice were randomly divided into the model group (*db/db*) and the AS-IV group (*db/db*+AS-IV) (n = 6 per group). AS-IV was added to standard feed at a dose of 1g/kg. The mice’s condition was checked every 2 days, and none of the animals died before reaching the experimental endpoint. After 12 weeks of AS-IV treatment, all mice were anesthetized (sodium pentobarbital, 75mg/kg, intraperitoneal injection), and blood samples were obtained by eye enucleation. The mice were euthanized by cervical dislocation without regaining consciousness. The kidneys were rapidly harvested and processed. One of the kidneys was used for mitochondrial isolation, while the other one was preserved for histological examination, Western blotting, and immunohistochemistry analysis.

### Blood glucose and urine index analysis

Blood glucose levels were determined using the Accu-Chek Performa blood glucose monitoring system (Roche, Germany) from blood samples obtained from the cut tip of the tail. Urinary albumin concentrations were measured using a mouse albumin ELISA quantitation set (Bethyl Laboratories, USA) according to the manufacturer’s protocol. Urine N-acetyl-β-D-glucosaminidase (NAG) was measured using the automated colorimetric method.

### Isolation of mitochondria from kidney

The kidneys were rapidly harvested after cervical dislocation and placed in plates filled with ice-cold isolation buffer-1 (IB-1) (225mmol/l mannitol, 75mmol/l sucrose, 5mmol/l HEPES, 2mmol/l K_2_HPO_4_, 1mmol/l EGTA, 0.1% bovine serum albumin [BSA], pH 7.2). The kidney cortex was isolated and cut into small pieces on the plate and transferred to a pre-cooled homogenizer loaded with 10 ml of IB-1. The kidney cortex pieces were then ground into homogenate on ice using a glass pestle. The homogenate was transferred to a pre-cooled 15-ml polypropylene centrifuge tube and centrifuged at 1,300*g*, 5 min at 4°C, and the supernatant fraction was gently suspended in ice-cold IB-2 (85% IB-1, 15% Percoll) in another two pre-cooled 10-ml polypropylene ultracentrifuge tubes and centrifuged at 17,000*g*, 18 min at 4°C. The supernatant was discarded, and the pellet was gently resuspended in 10-ml ice-cold IB-3 (225mmol/l mannitol, 75mmol/l sucrose, 5mmol/l HEPES, 2mmol/l K_2_HPO_4_, pH 7.2) and centrifuged at 10,000*g*, 10 min at 4°C. The final mitochondrial pellet was dissolved in IB-4 (99% IB-3, 1% protease inhibitor), and the protein concentration of mitochondrial suspension was measured in a Bradford protein assay (Bio-Rad, USA).

### Histological examination

For light microscopic analysis, mouse kidneys were fixed with 4% buffered paraformaldehyde (pH 7.4) at 4°C overnight, dehydrated in graded alcohols, and embedded in paraffin. The paraffin-embedded kidneys were cut into 4-μm sections and stained with periodic acid-Schiff (PAS) for the evaluation of pathologic changes. Twenty five glomeruli and approximately 80–100 proximal tubules in each mouse and six mice in each group were measured for mesangial matrix fraction and tubular area using Nikon NIS-Elements BR software version 4.10.00 (Nikon, Japan) by a pathologist in a blinded manner.

### Immunohistochemistry analysis

The paraffin-embedded kidneys were cut into 6-μm sections and dewaxed and rehydrated. After 20-minute antigen retrieval in 10 mM sodium citrate (pH 6.0), the sections were incubated with 3% hydrogen peroxide for 10 minutes at room temperature and then blocked with 10% goat serum for 1 hour at 37°C. The sections were stained with anti-Fis-1 (1:50), anti-PINK1 (1:100), and anti-Parkin (1:50) primary antibody at 4°C overnight followed by MaxVision^TM^ HRP-Polymer anti-Mouse/Rabbit IgG complex (Maixin Biotech, China) for 15 min at room temperature. The sections were then treated with diaminobenzidine (DAB) substrate (Maixin Biotech, China), followed by counterstaining with hematoxylin and examination. In all cases, antibody negative controls were evaluated to ensure that the results were not a consequence of cross-reactivity or nonspecific binding of the secondary antibodies.

### Immunofluorescence analysis

The paraffin-embedded kidneys were cut into 6 μm sections and dewaxed and rehydrated. After 3-hour antigen retrieval in 10 mM sodium citrate (pH 6.0), the sections were incubated with block buffer (5% BSA in PBS) for 1 hour at room temperature. The sections were stained with primaries antibodies at 4°C overnight followed by appropriate Alexa Fluor 488-conjugated IgG or Alexa Fluor 546-conjugated IgG secondary antibodies. To identify nuclei, tissues were counterstained with the fluorescent dye 4',6-diamidino-2-phenylindole (DAPI) for 3 minutes. In all cases, antibody negative controls were evaluated to ensure that the results were not a consequence of cross-reactivity or nonspecific binding of the secondary antibodies. All images were captured using a fluorescence microscope (Nikon, Japan).

### Western blotting

The frozen kidney cortex was cut into pieces under dry ice and was then homogenized in ice-cold cellular lysis buffer (150 mM NaCl, 10 mM Tris–HCl, 5 mM EDTA, 1 mM EGTA, and 10% Triton X-100) containing a protease inhibitor cocktail. The homogenates were sonicated for 15 seconds. The tissue lysates were centrifuged for 15 min at 8,000*g* at 4°C. The tissular and mitochondrial protein concentrations were measured in a Bradford protein assay. Equal amounts of total protein from the tissue lysates or isolated mitochondria were loaded and electrophoresed through 10% or 12% NuPAGE^®^Novex^®^ Bis-Tris pre-cast polyacrylamide gels (Thermo Fisher Scientific, USA) and were then transferred to polyvinylidene difluoride membranes (Millipore, USA). After being blocked in 5% non-fat milk for 1 hour at room temperature, the membranes were incubated with rabbit anti-PGC-1α (1:2000 dilution), rabbit anti-Drp-1 (1:1000 dilution), rabbit anti-Fis-1 (1:200 dilution), mouse anti-OPA-1(1:1000 dilution), rabbit anti-Mfn-2 (1:1000 dilution), rabbit anti-PINK1 (1:500 dilution), mouse anti-Parkin (1:1000 dilution), rabbit anti-Parkin (phospho S65) (1:500 dilution), rabbit anti-VDAC (1:1000 dilution), mouse anti-Mfn-1 (1:2000 dilution), rabbit anti-NRF-1 (1:1000 dilution), rabbit anti-MFF (1:500 dilution), rabbit anti-LC-3 (1:1000 dilution), and mouse anti-β-actin (1:4000 dilution) antibody at 4°C overnight. Then, the membranes were incubated with the appropriate HRP-conjugated secondary antibodies for 1 hour at room temperature. HRP activity was visualized using Clarity^TM^ Western ECL Substrate and a ChemiDoc^TM^ MP Imaging System (Bio-Rad Laboratories, USA). The densitometric analysis was performed using Image Lab^TM^ software version 5.1 (Bio-Rad Laboratories, USA).

### Statistical analysis

The data are presented as the means ± SEM. The differences among groups were analyzed for statistical significance by one-way ANOVA followed by *post hoc* analysis using the Least Significant Difference (LSD) test if equal variances were assumed or the Games-Howell test if equal variances were not assumed. A value of *P*<0.05 was considered statistically significant. All statistical analyses were performed using SPSS statistics software (version 16.0, SPSS Inc., Chicago, IL, USA).

## Results

### AS-IV reduced urinary albumin excretion (UAE) in *db/db* mice

**Fig 2:**
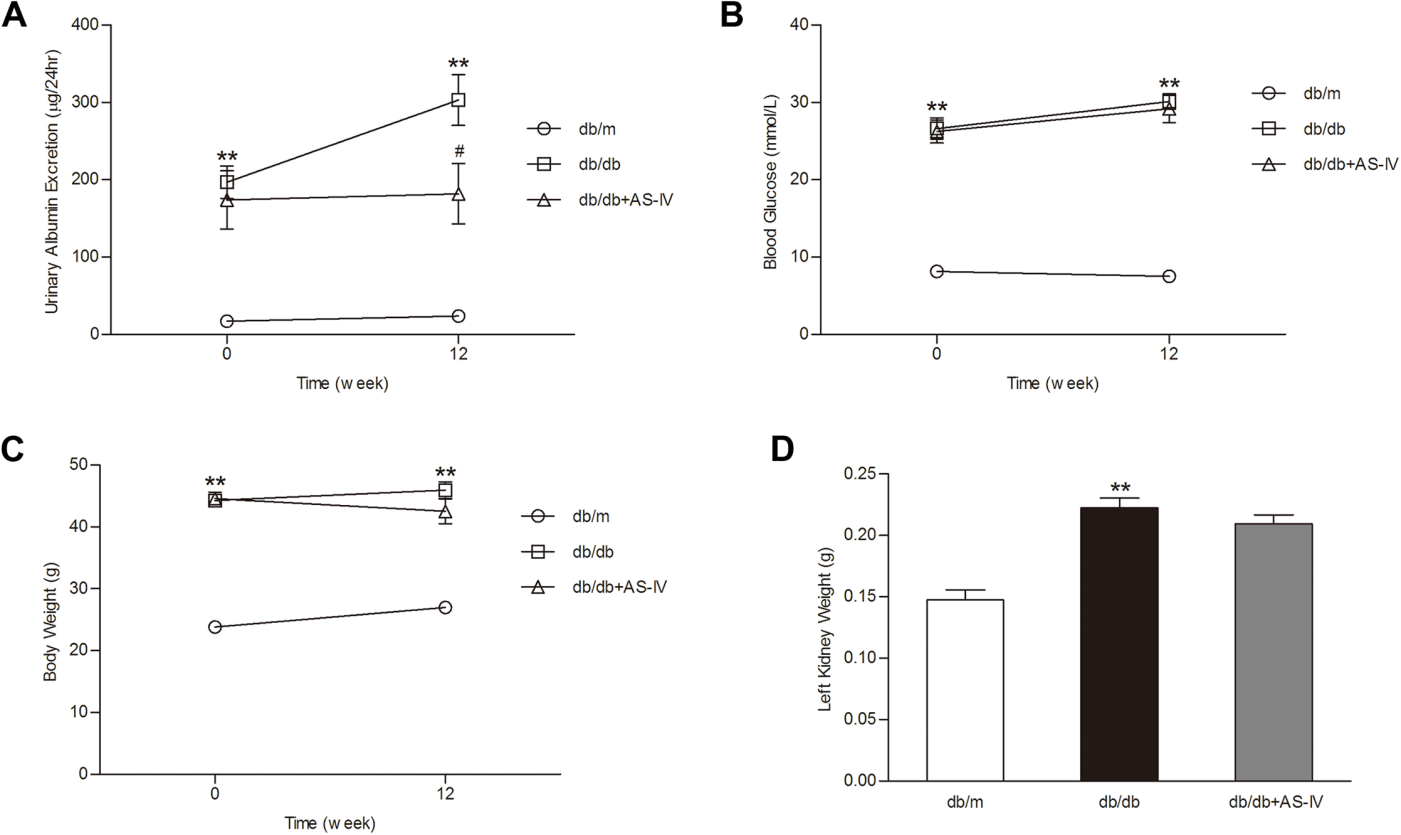
Effect of AS-IV on physiological parameters in *db/db* mice. **(A)** AS-IV administration significantly reduced UAE in *db/db* mice. There were no significant differences of blood glucose level **(B)**, body weight **(C)**, and kidney weight **(D)** between the *db/db* group and the *db/db*+AS-IV group. Data are presented as the means ± SEM, n = 6 mice per group. (**P<0.01 compared with *db/m* group). AS-IV: Astragaloside IV; UAE: urinary albumin excretion.

As shown in Fig 2A, AS-IV administration significantly reduced UAE in *db/db* mice at week 12 (182±39μg/24hr *vs*. 303±33μg/24hr, *P*<0.05). However, the comparisons of blood glucose, body weight, and kidney weight between the *db/db* group and the *db/db*+AS-IV group were not significant (Fig 2B and 2C). These data suggested that the effect of reducing UAE of AS-IV is independent of reductions in blood glucose and body weight.

### AS-IV ameliorated renal injury in *db/db* mice

**Fig 3:**
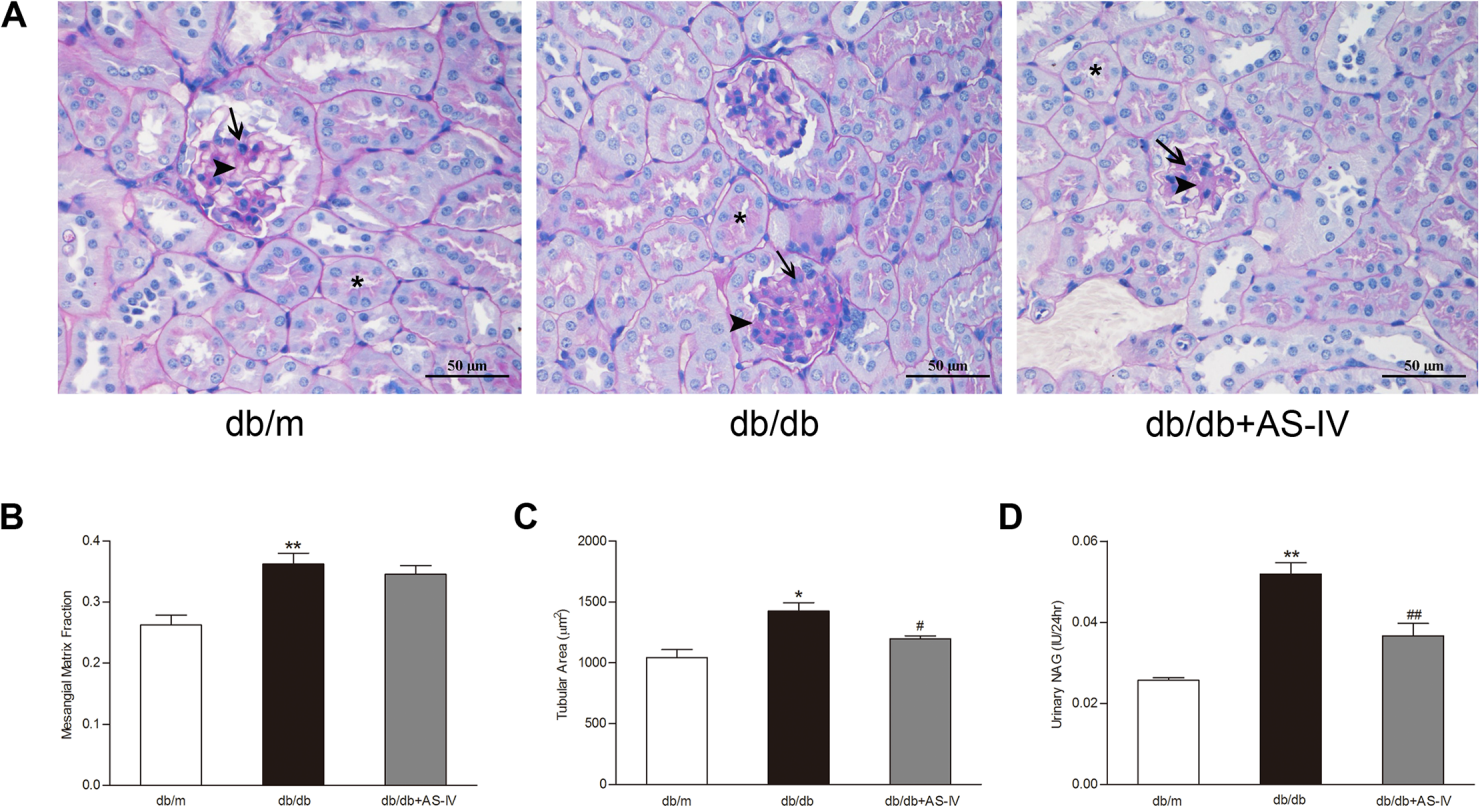
AS-IV ameliorates renal injury in *db/db* mice. **(A)** PAS staining showing representative images of renal tissue from all groups. All images are shown at identical magnification, ×400. Asterisk indicates proximal tubule, arrow indicates mesangial cell, arrowhead indicates mesangial matrix. **(B)** Quantitative analysis of glomerular mesangial matrix fraction. **(C)** Quantitative analysis of proximal tubular area according to outer diameter. **(D)** AS-IV administration reduced excess excretion of urinary NAG. The data are presented as the means ± SEM, n = 6 mice per group. (^*^P<0.05, ^**^P<0.01 compared with the *db/m* group; ^#^*P*<0.05, ^##^*P*<0.01 compared with the *db/db* group). AS-IV: Astragaloside IV; NAG: N-acetyl-β-D-glucosaminidase.

Fig 3A showed the PAS-stained sections of kidney from all groups. In the quantitative analyses, we found significant increase of glomerular mesangial matrix and proximal tubular area in the *db/db* group. Treatment of AS-IV slightly reduced glomerular mesangial matrix and significantly reduced proximal tubular area in the *db/db*+AS-IV group (Fig 3B and 3C). In addition, urinary NAG excretion, marker of proximal tubular injury, was also significantly increased in *db/db* mice, and could be restored by AS-IV treatement (Fig 3D).

### AS-IV downregulated mitochondrial fission-associated protein expression in *db/db* mice

**Fig 4:**
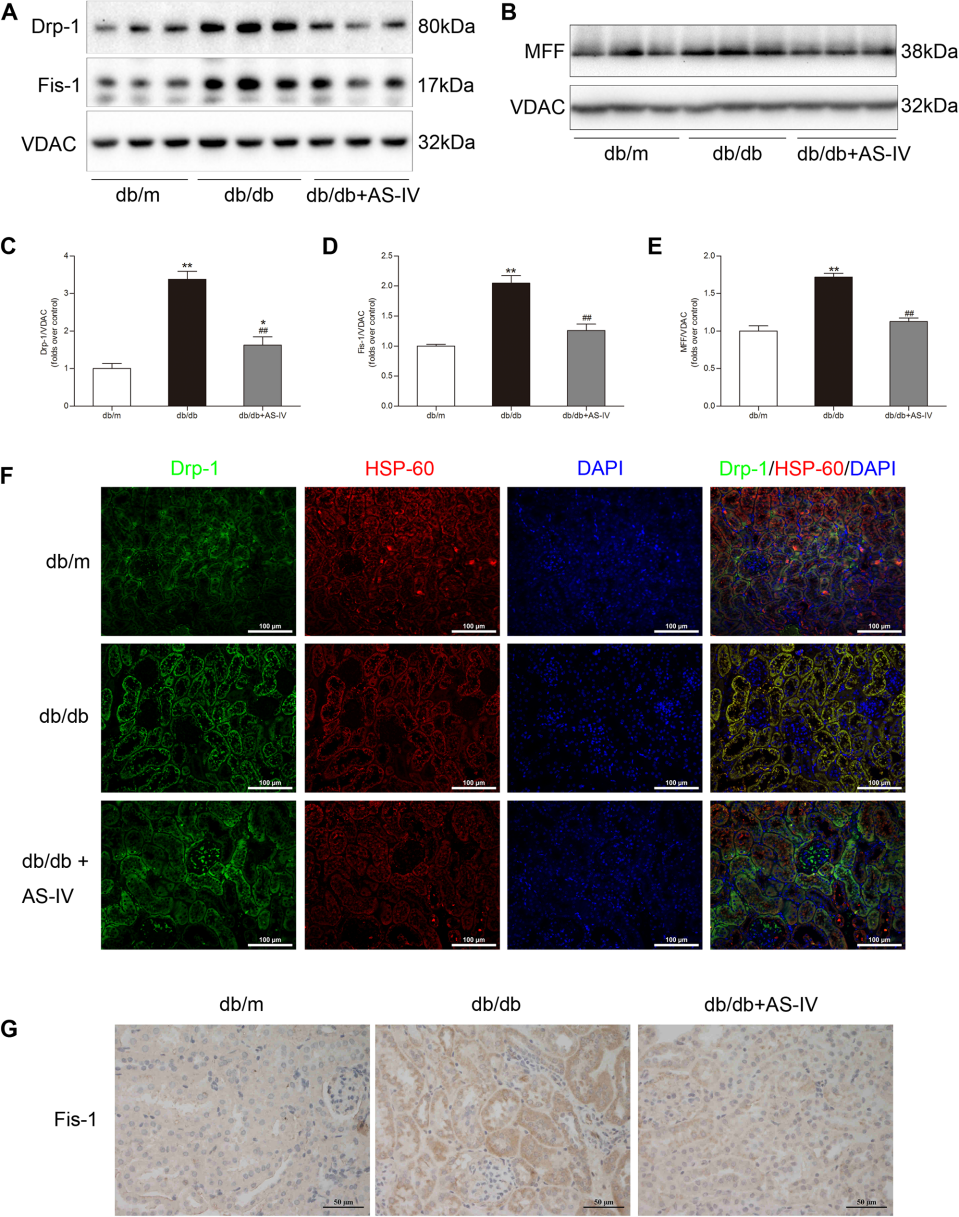
AS-IV downregulates mitochondrial fission-associated protein expression in *db/db* mice. **(A,B)** Representative Western blot images indicated that Drp-1, Fis-1, and MFF protein expression were significantly increased in the *db/db* group but were abolished by AS-IV treatment. **(C-E)** Densitometric analysis of Drp-1, Fis-1, and MFF protein expression normalized to VDAC content. **(F)** Representative immunofluorescence images of Drp-1 and HSP-60. **(G)** Representative immunohistochemistry images of Fis-1. All images are shown at identical magnification, ×200 (F) or ×400 (G). Data are presented as the means ± SEM, n = 6 mice per group. (^*^P<0.05, ^**^P<0.01 compared with the *db/m* group; ^##^*P*<0.01 compared with the *db/db* group). AS-IV: Astragaloside IV; DAPI: 4',6-diamidino-2-phenylindole; Drp-1: dynamin-related protein 1; Fis-1: mitochondrial fission protein 1; HSP-60: heat shock protein 60; VDAC: voltage-dependent anion channel.

Isolated kidney mitochondria were used for mitochondrial fission analysis. Western blotting revealed that the expression levels of Drp-1, Fis-1, and MFF, the main regulators of mitochondrial fission, were significantly upregulated in *db/db* mice. AS-IV administration restored the protein abundance of Drp-1, Fis-1, and MFF (Fig 4A–4E). Immunofluorescence analysis indicated that more Drp-1 co-localized with HSP-60, a mitochondrial marker, in the *db/db* group (Fig 4F). The expression of Fis-1 was confirmed by immunohistochemistry analysis (Fig 4G). The above data implied that AS-IV restrains the increase of mitochondrial fission in the kidneys of *db/db* mice.

### AS-IV had no effect on mitochondrial fusion and mitochondrial biogenesis-associated protein expression in *db/db* mice

**Fig 5:**
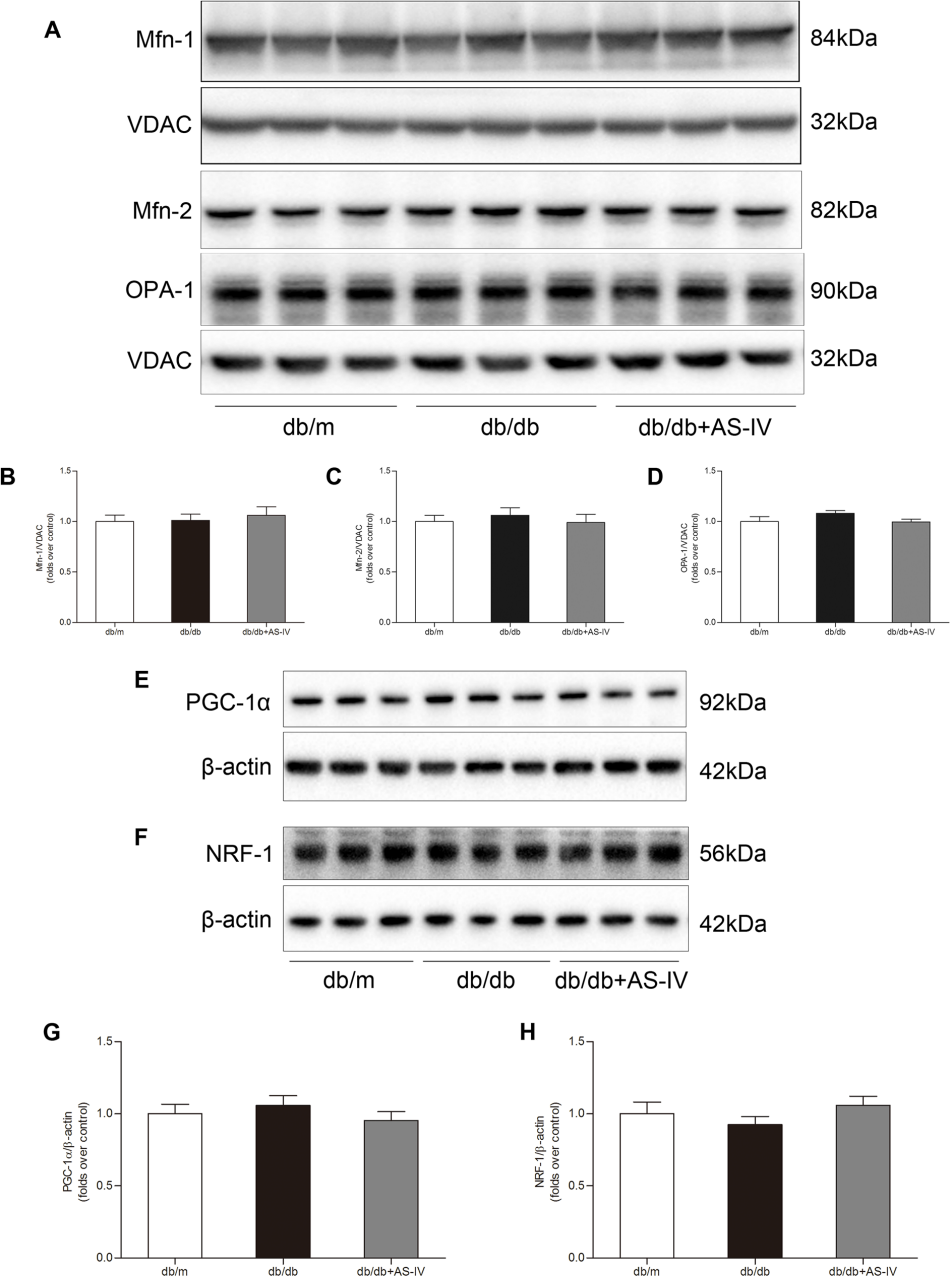
AS-IV has no effect on mitochondrial fusion and biogenesis-associated protein expression in *db/db* mice. **(A)** Representative Western blot images show that Mfn-1, Mfn-2, and OPA-1 protein expression levels were not significantly different among the three groups. **(B-D)** Densitometric analysis of Mfn-1, Mfn-2 and OPA-1 protein expression normalized to VDAC content. **(E,F)** Representative Western blot images show that PGC-1α and NRF-1 protein expression levels were not significantly altered in *db/db* mice with or without AS-IV treatment. **(G,H)** Densitometric analysis of PGC-1α and NRF-1 protein expression normalized to β-actin content. Data are presented as the means ± SEM, n = 6 mice per group. AS-IV: Astragaloside IV; Mfn-1: mitofusin 1; Mfn-2: mitofusin 2; NRF-1: nuclear respiratory factor 1; OPA-1: optic atrophy 1; PGC-1α: peroxisome proliferator-activated receptor-γ coactivator-1α, VDAC: voltage-dependent anion channel.

Mfn-1/2 and OPA-1 are separately responsible for outer and inner mitochondrial membrane fusion. In the present study, Mfn-1/2 and OPA-1 protein expression levels were not significantly different among the three groups (Fig 5A–5D). In addition, the protein abundance of PGC-1α, the master regulator of mitochondrial biogenesis, and its downstream signal NRF-1 were also not significantly altered in *db/db* mice with or without AS-IV treatment (Fig 5E–5H).

### AS-IV downregulated mitophagy-associated protein expression in *db/db* mice

**Fig 6:**
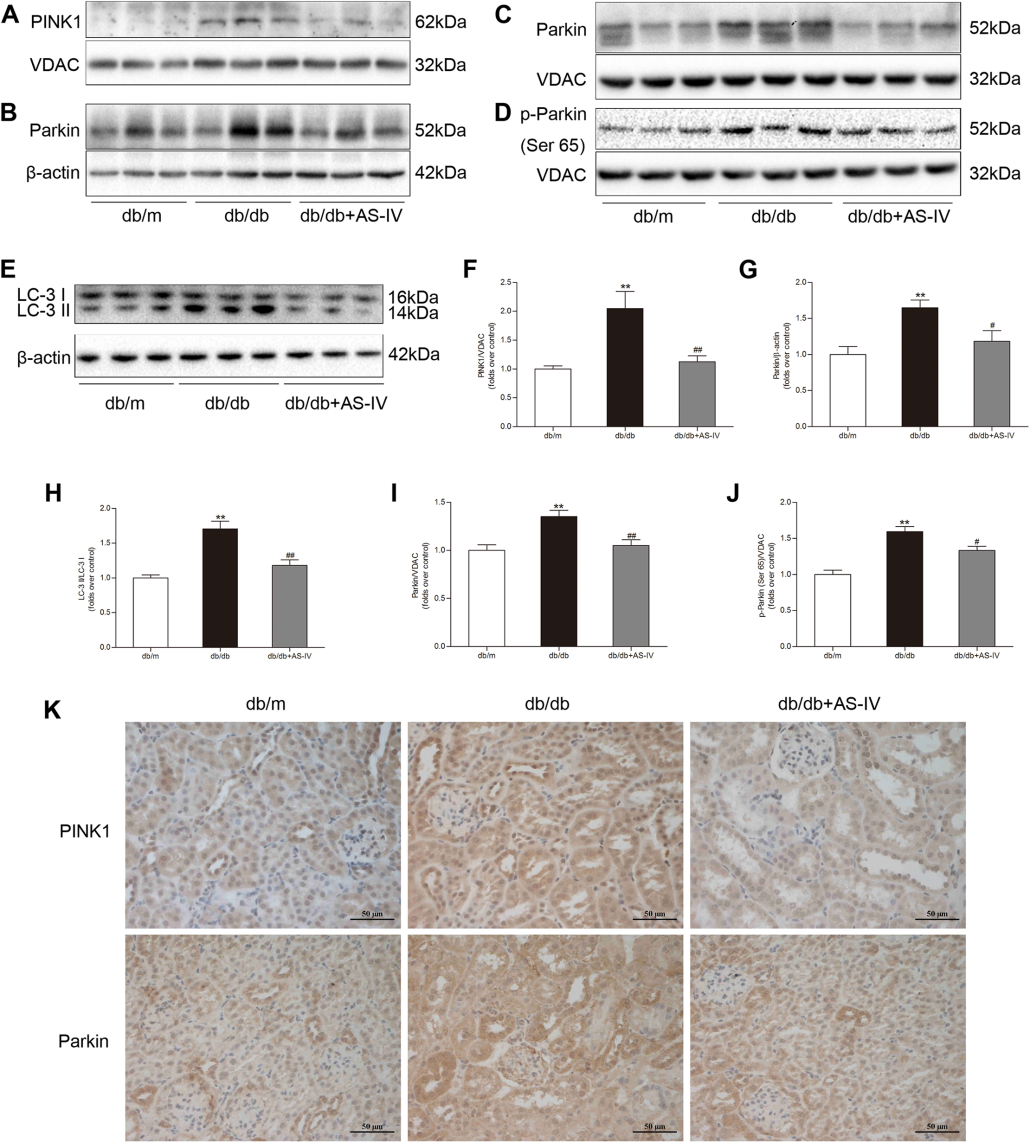
AS-IV downregulates mitophagy-associated protein expression in *db/db* mice. **(A-E)** Representative Western blot images showing significant increases of PINK1, Parkin, p-Parkin (Ser 65), and LC-3II protein expression in *db/db* mice. AS-IV administration significantly reduced the expression levels of these proteins. **(F-J)** Densitometric analysis of PINK1, Parkin, p-Parkin (Ser 65), and LC-3II protein expression normalized to VDAC or β-actin content. **(K)** Representative immunohistochemistry images of PINK1 and Parkin. All images are shown at identical magnification, ×400. Data are presented as the means ± SEM, n = 6 mice per group. (^**^P<0.01 compared with the *db/m* group; ^#^*P*<0.05, ^##^*P*<0.01 compared with the *db/db* group). AS-IV: Astragaloside IV; LC-3: light chain 3; PINK1: PTEN-induced putative kinase 1; VDAC: voltage-dependent anion channel.

PINK1/Parkin-mediated mitophagy is the best known mitophagy pathway. As shown in Fig 6A and 6F, PINK1 expression was overtly upregulated in the kidney mitochondria of *db/db* mice. PINK1 stabilization and accumulation in mitochondria would further recruit Parkin from the cytoplasm to the mitochondria and phosphorylate it at Serine 65. Western blotting showed that total Parkin in the kidney, Parkin translocated to the mitochondria and its active form, p-Parkin (Ser 65), were all upregulated in *db/db* mice (Fig 6B–6D, Fig 6G, 6I and 6J). These results were confirmed by immunohistochemistry analysis (Fig 6K). In addition, the protein level of LC-3II was also upregulated in the kidney of *db/db* mice (Fig 6E and 6H). However, AS-IV administration significantly restored PINK1, Parkin, p-Parkin (Ser 65), and LC-3II protein expression (Fig 6A–6K). Taken together, these data suggest that mitophagy-associated proteins were upregulated in *db/db* mice but abolished by AS-IV treatment.

## Discussion

In the present study, we examined the effect of AS-IV on retarding DN progression in an experimental animal model of type 2 diabetes, *db/db* mice. AS-IV administration significantly ameliorated the albuminuria and renal pathologic injury observed in diabetic *db/db* mice. These renoprotective effects of AS-IV were accompanied by restoration of the mitochondrial quality control network, which was deranged in *db/db* mice.

The role of mitochondria in the pathophysiology of renal disease is gaining increasing attention [13]. The mitochondrial quality control network has been investigated in various diseases, but in kidney diseases, including DN, the investigations are limited. Most previous studies regarding mitochondrial quality control in DN employed streptozotocin (STZ)-induced type 1 diabetic mice, and few of these studies used an animal model of type 2 diabetes. Considering different physiopathologic mechanisms of type 1 and type 2 diabetes, it is reasonable to believe that distinct mitochondrial quality control statuses exist in different types of diabetic kidneys. In our study, we noted some changes in the mitochondrial quality control network in *db/db* mice that were different from the results of previous studies in STZ-induced mice.

PGC-1α is a master regulator of mitochondrial biogenesis and is known to co-activate nuclear respiratory factor 1/2 (NRF-1/2). The NRFs, in turn, activate mitochondrial transcription factor A, which is directly responsible for transcribing nuclear-encoded mitochondrial proteins [14]. Recently, Sharma *et al*. found that PGC-1α mRNA expression was reduced within microdissected cortical tubulointerstitial samples from patients with diabetic kidney disease arising from type 1 (n = 12) or type 2 (n = 49) diabetes [15]. In an animal model, Xiao *et al*. found that PGC-1α mRNA and protein levels were notably decreased in STZ-induced rat kidneys compared with control subjects [16]. In the present study, the protein levels of both PGC-1α and its downstream signal, NRF-1, were not significantly altered in the kidney cortexes of *db/db* mice, with or without AS-IV treatment, compared with *db/m* mice. Mitochondria are highly dynamic organelles, constantly undergoing fission and fusion. A recent study by Zhan *et al*. showed that the mitochondrial pro-fission proteins Drp-1 and Fis-1 were upregulated in the renal tubules of STZ mice, whereas the mitochondrial pro-fusion protein Mfn-2 was decreased in STZ mice [17]. Similarly, our results showed that Drp-1 and Fis-1 were increased in the kidneys of *db/db* mice. However, Mfn-2 and OPA-1expression were not significantly changed in *db/db* mice. AS-IV administration abolished increased Drp-1 and Fis-1 expression in *db/db* mice, which might explain its renoprotective effect. In accordance with this finding, another study found that conditional podocyte-specific deletion of Drp-1 improved mitochondrial fitness and protected against DN progression in *db/db* mice [18]. Mitophagy is the selective degradation of mitochondria by autophagy that promotes the turnover of mitochondria and prevents the accumulation of dysfunctional mitochondria, which could lead to cellular degeneration. Similar to autophagy, mitophagy could serve to protect cells but might also contribute to cell damage [19]. PINK1/Parkin signal is the best characterized pathway of mitophagy in mammalian cells [20]. An emerging body of evidence indicates that autophagy is involved in DN progression [21]; however, less is known about the role of mitophagy in DN. In a rat model of type 1 diabetes, PINK1 protein expression was increased in the renal cortex after 4 weeks of STZ injection [22]. In contrast, Zhan *et al*. showed that PINK1 protein was downregulated in the renal cortex of ICR mice after 8 weeks of STZ injection [17]. In our study, we found that the protein levels of PINK1, Parkin, and phosphorylated Parkin at Ser 65 were all increased in the renal mitochondria of 21-week-old *db/db* mice. In addition to being a different study design, a plausible explanation for these conflicting results is that the level of mitophagy changes with DN progression.

AS-IV is the main active substance of *Astragalus membranaceus Bunge*, which has been used to treat numerous diseases for thousands of years. AS-IV has multiple pharmacologic effects, including anti-inflammatory, anti-fibrotic, anti-oxidative stress, anti-asthma, anti-diabetes, and immunoregulatory effects [8]. The renoprotective effect of AS-IV in the STZ-induced animal model has been clarified in previous studies, which are associated with podocyte protection [11,12], anti-inflammation [9], and inhibition of endoplasmic reticulum stress [10]. Our study confirmed the renoprotective effect of AS-IV in DN in a type 2 diabetic mouse model, *db/db* mice. A previous study found that AS-IV could prevent amyloid beta1-42-induced neurotoxicity in SK-N-SH cells by inhibiting mitochondrial permeability transition pore opening and mitochondrial superoxide generation to maintain the function of mitochondria [23]. For the first time, our data suggest that AS-IV contributes to the restoration of the mitochondrial quality control network, which might be associated with its renoprotective effect. However, in present study we did not evaluate the effect of AS-IV on *db/m* mice, not investigate how mitochondrial quality control contributes to DN and how AS-IV confers protective effects on DN. Further studies are needed to elucidate these questions.

In conclusion, 21-week-old *db/db* mice show overt renal injury accompanied by derangement of the mitochondrial quality control network. AS-IV administration significantly ameliorates renal injury in *db/db* mice, and this renoprotective effect might be exerted by restoration of the mitochondrial quality control network.
